# A Systemic Prime–Intrarectal Pull Strategy Raises Rectum-Resident CD8+ T Cells for Effective Protection in a Murine Model of LM-OVA Infection

**DOI:** 10.3389/fimmu.2020.571248

**Published:** 2020-09-24

**Authors:** Qian He, Lang Jiang, Kangli Cao, Linxia Zhang, Xinci Xie, Shuye Zhang, Xiangqing Ding, Yongquan He, Miaomiao Zhang, Tianyi Qiu, Xuanxuan Jin, Chen Zhao, Xiaoyan Zhang, Jianqing Xu

**Affiliations:** Shanghai Public Health Clinical Center and Institutes of Biomedical Sciences, Shanghai Medical College, Fudan University, Shanghai, China

**Keywords:** mucosal immune response, rectum TRM, prime–pull vaccination strategy, HIV, *Listeria monocytogenes*

## Abstract

As the entry sites of many pathogens such as human immunodeficiency virus (HIV), mucosal sites are defended by rapidly reacting resident memory T cells (TRM). TRMs represent a special subpopulation of memory T cells that persist long term in non-lymphoid sites without entering the circulation and provide the “sensing and alarming” role in the first-line defense against infection. The rectum and vagina are the two primary mucosal portals for HIV entry. However, compared to vaginal TRM, rectal TRM is poorly understood. Herein, we investigated the optimal vaccination strategy to induce rectal TRM. We identified an intranasal prime–intrarectal boost (pull) strategy that is effective in engaging rectal TRM alongside circulating memory T cells and demonstrated its protective efficacy in mice against infection of *Listeria monocytogenes*. On the contrary, the same vaccine delivered via either intranasal or intrarectal route failed to raise rectal TRM, setting it apart from vaginal TRM, which can be induced by both intranasal and intrarectal immunizations. Moreover, intramuscular prime was also effective in inducing rectal TRM in combination with intrarectal pull, highlighting the need of a primed systemic T cell response. A comparison of different pull modalities led to the identification that raising rectal TRM is mainly driven by local antigen presence. We further demonstrated the interval between prime and boost steps to be critical for the induction of rectal TRM, revealing circulating recently activated CD8+ T cells as the likely primary pullable precursor of rectal TRM. Altogether, our studies lay a new framework for harnessing rectal TRM in vaccine development.

## Introduction

For many human disease-causing pathogens, the first step toward the establishment of a successful infection is to cross the mucosal barrier before getting access to the underlying tissues and, in some cases, further to the systemic circulation. Thus, an effective immune response against these pathogens constitutes two components: the mucosal response majoring in rapidly detecting the invading pathogens and containing them locally and the systemic immunity, which provides the later enforcement if the frontline mucosal defense is breached (1). This two-layer defense concept and its implication in vaccine development has been excellently illustrated by the yet unsolved HIV-1 challenge.

HIV-1 has infected more than 70 million people worldwide and resulted in ~35 million deaths. Despite extensive efforts to find effective HIV vaccines, including several large-scale clinical trials with both T-cell- (2, 3) and antibody-based vaccines (4), very little success has been attained, with RV144 being the only vaccine showing encouraging results with a moderate 31.2% protective efficacy. Given that all of the clinically tested vaccines so far were aiming to raise systemic immunity against HIV-1, their inefficacy called into question whether systemic antibody and T cell response are sufficient to confer adequate protection against HIV-1 infection. A reasonable postulation is that systemic immunity, coming into effect after local virus replication, might be “too late and too little” for containing the HIV viruses once they overcome the portal barrier to establish systemic infection (5, 6).

The central member of mucosal immunity is a unique subset of memory T cells, namely, resident memory T cell (TRM). The TRM designation originates from the long-lasting local lodgment of these cells without attendance in the systemic circulation. Unlike their circulating memory T cell counterparts, TRMs serve as the sentinel setting to guard their habitat against the invasion of harmful foreign pathogens (7, 8). They continuously patrol their periphery non-lymphoid niche and respond rapidly to local pathogen re-encounter by either direct lysis of infected cells or production of interferon-γ (IFN-γ) and inflammatory chemokines, which consequently result in the timely recruitment of circulating lymphocytes (e.g., memory CD8+ T cells, B cells, and innate leukocytes) to the site of infection to facilitate viral clearance (9–12).

In this study, we are focusing on rectum TRM, which is one of the least understood TRM despite of potential significant role in HIV infection. HIV viruses can be contracted either through vagina or rectum, while there is growing evidence supporting men having sex with men (MEM) as the major human population at highest risk for HIV infection (13, 14). On the other hand, animal studies using non-human primates have already pointed to the importance of vagina (15) and rectum (16, 17) TRMs in the clearance or containment of HIV infection. Rectal TRM, albeit potentially with the same importance as vaginal TRM for a T-cell-based HIV vaccine to be protective, has been less studied as compared to TRM of vagina and other mucosal sites. To date, there has been no consensus on the best strategy to induce vaginal TRM. Several studies claimed that vaginal TRM could be elicited by intranasal immunization (18, 19), in line with the long-standing notion of “common mucosal immune system,” which implicates that the immune response at a mucosal site can be raised by the administration of antigen to a distant mucosal site. More complicated vaccination regimens have also been explored, the most notable of which is the prime–pull strategy first developed by Shin Shin and Iwasaki. This strategy consists of two steps: a parenteral vaccination to raise systemic cellular response (prime), followed by a vaginal delivery of chemokine or vaccine that directs the tissue targeting of the prime-activated circulating T cells (pull) (20). As for the induction of rectal TRM, previous studies mainly explored the intrarectal route, showing that repeated rectal vaccination led to a modest rectum TRM response (16, 17). Thus, although the vagina TRM studies provide useful lessons regarding the potential vaccination strategy for the induction of rectal TRM, the actual value of these lessons is uncertain and contingent on whether the recruitment and retention of T cells to the vagina and rectum share the same homing mechanism.

Here, we conducted a comprehensive investigation of vaccination strategies to elicit protective rectal TRM in mice. This resulted in the identification of a sequential vaccination strategy composed of intranasal administration (prime) followed by intrarectal inoculation (pull) as the optimal strategy to raise a vigorous rectal TRM response. Importantly, this response contributed largely to an effective protection against infection of *L. monocytogenes*. We further identified the newly activated CD8+ T cells as the primary source of rectal TRM, highlighting the impact of the interval between priming and pulling steps on the efficacy of vaccine regimen in eliciting these TRM cells. Collectively, our study sheds new light on the basic biology of rectal TRM and opens a new avenue for harnessing these cells in vaccine development.

## Materials and Methods

### Mice and Treatment Reagents

Wild-type naive mice were 6- to 8-week-old female C57BL/6 mice purchased from the B&K Universal Group Ltd. (Shanghai, China). OT-I mouse, which harbors transgenic T cell receptor recognizing ovalbumin residues 257–264 in the context of H2Kb, was obtained from Jackson Laboratory. All mice were maintained under specific pathogen-free (SPF) conditions at the animal facilities of Shanghai Public Health Clinical Center, Fudan University (Shanghai, China). Purified ovalbumin (OVA) protein was purchased from Sigma (Sigma, Cat. No. A5378) and dissolved in sterile normal saline. Recombinant Tiantan vaccinia virus encoding OVA (rTTV-OVA) was previously described (21). Recombinant influenza virus vector H9N2 expressing OVA_257−264_ (H9N2-OVA_257−264_) (22) was kindly provided by Prof. Zejun Li (Shanghai Veterinary Research Institute). *L. monocytogenes* expressing OVA (LM-OVA) with streptomycin resistance (23) was a generous gift from Prof. Jianhua Li (Key Laboratory of Medical Molecular Virology, Department of Medical Microbiology, Shanghai Medical College, Fudan University). The CXCL10 chemokines were purchased from Novus, and cholera toxins (CT) were purchased from Sigma. FTY720 used for blocking circulating T cells was purchased from Cayman Chemical.

### Adoptive Transfer

For single adoptive transfer, CD8+ OT-I cells were isolated from the spleen of OT-I mice using mouse CD8+ T cell negative isolation kit (Stem cell, Cat. No. 19853) and transferred to recipient mice by intravenous injection at 2 × 10^5^/mouse). For the isolation of the first generation of adoptively transferred OT-I cells from tissues in the successive adoptive transfer protocol as described in **Figure 5**, we used two different magnetic cell selection methods: For the spleen, blood, and iliac LN, we first enriched the CD8+ T cells using mouse CD8+ T cell negative isolation kit (stem cell, Cat. No. 19853) and then isolated the CD45.1+ T cells using the Miltenyi isolation kit (Cat. No. 130-048-801, 130-042-401). For the lung, we isolated the CD45.1+ T cells directly using the Miltenyi isolation kit.

### Immunization and Infection of Mice

Where indicated, the mice were primed or boosted intranasally (IN), intramuscularly (IM), or intrarectally (IR), denoted respectively as IN, IM, and IR. The volume of formulation given IN or IM was 50 μl, and IR was 20 μl in phosphate-buffered saline (PBS). For the vaccination with protein immunogens, OVA in this study, 10 μg of protein was injected together with indicated adjuvants. The amounts of adjuvant used were 1 μg for CT, 3 μg for CXCL10, and 1:1 volume mixing with immunogen for alum. In the case of immunization using the H9N2-OVA_257−264_ virus, mice were anesthetized and intranasally (IN) inoculated with 1000 TCID50 H9N2-OVA_257−264_. For using rTTV-OVA, the intrarectal (IR) infection was performed at a dose of 2 × 10^6^ plaque forming unit (PFU) per mice. The detailed vaccination schedules and regimens are described in section Results.

**Figure 1:**
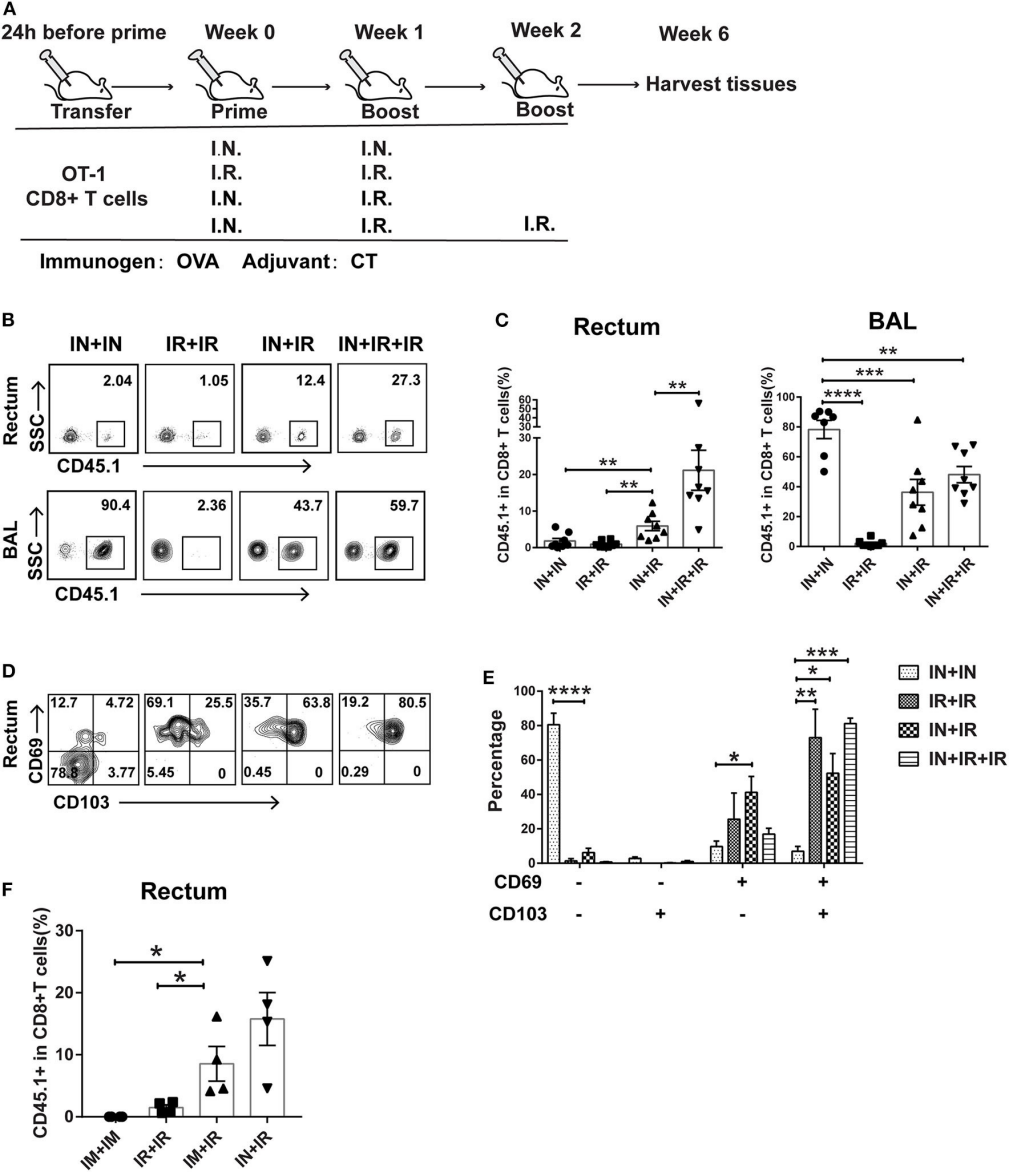
Intranasal and intrarectal routes needed to be combined for optimal induction of rectum-resident memory T cell (TRM). **(A)** Experimental scheme. For all the regimens tested, OVA + CT was used for both priming and boosting events. IN, intranasal; IR, intrarectal. **(B)** Representative flow cytometry plots of detection of CD45.1+ CD8+ T cells in rectum and lung bronchoalveolar lavage (BAL) of vaccinated mice. **(C)** Pooled data showing the frequencies of CD45.1+ CD8+ T cell as percentages of total CD8+ T cells in the rectum (left panel) and BAL compartments (right panel) (*n* = 8). **(D)** Representative flow cytometry plots of CD69 and CD103 expression of CD45.1+ CD8+ T cells in the rectum of vaccinated mice. **(E)** Pooled data showing the percentage of CD69+ and/or CD103+ cells in CD45.1+CD8+ T cells in the rectum (*n* = 4). **(F)** Mice were either intranasally (IN) or intramuscularly (IM) primed prior to intrarectal (IR) boosting, following the same schedule shown in **(A)**; 4 weeks later, mice were sacrificed for immune response analysis. Pooled data showing the frequencies of CD45.1+ CD8+ T cell in total CD8+ T cells in the rectum. Bars showed the mean ± SEM pooled from animals of the same experimental group. *p < 0.05, **p < 0.01, ***p < 0.001, ****p < 0.0001, ns: *p* > 0.05. Data are representative of three independent experiments.

### Tissue Preparation

At indicated time postimmunization or Listeria-OVA challenge, mice were sacrificed, and spleens, iliac lymph nodes, bronchi alveolar lavage (BAL) fluids, and rectums were immediately harvested. For lung preparation, the lungs were perfused using 5 ml of PBS injected in the right ventricle and welled out from the cut of the left atrium. The lung and rectum isolated were digested with 0.5 mg/ml of type I collagenase (Sigma, Cat. No. SCR103) for the lung and 0.5 mg/ml of the type II collagenase (Sigma, Cat No. C6885) for rectum (shaking 60 min at 37°C, 300 rpm) prior to mechanical dissociation through a 70-mm filter. The lymphocytes contained in the resulting rectum homogenates were then isolated with mouse 1 × lymphocyte separation, Medium (Dayou, Cat. No. DKW33-R0100).

### Flow Cytometry

The freshly isolated splenocytes, lymphocytes, or BAL cells were stained for 20 min at room temperature using the following fluorochrome-labeled specific antibodies: Alexa Fluor 700 antimouse CD3 (Clone: 17A2; BD), antigen-presenting cell (APC)-labeled antimouse CD8 (Clone: 53-6.7; BD), fluorescein isothiocyanate (FITC)-labeled antimouse CD45.1 (Clone: A20; Biolegend), phycoerythrin (PE)-labeled antimouse CD69 (Clone: H1.2F3; BD), PerCP-Cy5.5-labeled antimouse CD103 (Clone: M290; Biolegend), Brilliant Violet 421-labeled antimouse CXCR3 (Clone: CXCR3-173; Biolegend), FITC-labeled antimouse CD44 (Clone: IM7; BD), and PE-labeled antimouse LPAM-1(α4β7) (Clone: DATK32; BD). A viability dye (Life Technologies) was also included in the staining mix to differentiate living and dead cells. The stained samples were subjected to running on BD LSRFortessa^TM^ instrument followed by analysis with FlowJo X software (Tree Star, Inc.).

### Immunofluorescence

Harvested rectums were fixed in 8% paraformaldehyde for 2 h, treated with 30% sucrose overnight, and then subjected to optimal cutting temperature (OCT) embedding with liquid nitrogen. The resulting frozen tissue blocks were processed, stained, and imaged by TissueFAX (TissueGnostics, Austria). The primary antibodies used for staining included mouse anti-CD45.1 antibody (Clone: A20; Arigo Biolaboratories) and rat anti-CD8 antibody (Abcam, No. YTS169.4); the secondary antibodies were goat antimouse immunoglobulin G (IgG) (H + L), Alexa Fluor 488 (Invitrogen, No. A28175), and goat antirat IgG (H + L), Alexa Fluor 647 (Invitrogen, No. A21247). Nuclei were detected by incubation with 4′,6-diamidino-2-phenylindole dihydrochloride (DAPI).

### IFN-γ ELISPOT Assay

Enzyme-linked immunosorbent spot (ELISPOT) assays for IFN-γ release were performed using mouse IFN-γ ELISPOT kit (BD Bioscience) as previously described (21). In brief, a total of 2 × 10^5^ freshly isolated splenocytes or tissue-associated lymphocytes were plated in six replicates in 96-well plates precoated with purified antimouse IFN-γ. Three replicates were incubated for 24 h with immunogen-specific peptide or peptide pools. When OVA was used as the immunogen, a mouse H-2Kb-matched OVA peptide (OVA_257−264_: SIINFEKL) was adopted. After peptide stimulation, the cells were removed, and the plates were extensively washed with PBS/0.05% Tween before the addition of biotinylated anti-IFN-γ antibody (100 μl/well of 1 μg/ml). After overnight incubation at 4°C, avidin horseradish peroxidase (avidin-HRP) was added to the plates following wash steps with PBS, and the plates were subsequently developed according to the manufacturer's manual. Spots representing IFN-γ-producing cells were enumerated using an ELISPOT reader (ChampSpot III Elispot Reader, Saizhi, Beijing, China). The final value was calculated by subtracting the background value from the measured values.

### ELISA for IgG and IgA to OVA

The serum and rectal lavage antibodies against OVA were assessed using an enzyme-linked immunosorbent assay (ELISA) as previously described (24). Briefly, 96-well EIA/RIA plates were coated overnight with 1 μg/ml of OVA protein at 4°C. After removal of OVA protein and blocking with 5% milk, serially 2-fold diluted test samples were added to the wells. The bound antibodies were subsequently detected by incubation with 1:2,000 diluted goat antimouse IgG (HRP labeled) (Santa Cruz Biotechnology, Cat. No. sc-2005) or goat antimouse IgG (HRP labeled) (Santa Cruz Biotechnology, Cat. No. sc-2005) followed by development with OPD substrate (Thermo Scientific, Cat No. 34006) for measurement at 450 nm. The endpoint of serum antibody titer was determined as the reciprocal of the highest dilution that gives absorbance value of 2 standard deviations above the negative control.

### Intrarectal Infection Challenge

The intrarectal *Listeria*-OVA challenging was performed at two different dosages depending on the experimental purpose: 1 × 10^9^ CFU for the sublethal challenge and 5 × 10^9^ CFU for the lethal challenge. The infected animals were daily monitored for weight changes and mortality. For the detection of bacteria loads, the rectum was harvested from infected mice 3 days postchallenge, homogenized, and the resulting homogenate was serially diluted and plated on brain heart infusion agar plates supplemented with 50 μg/ml streptomycin. Colonies were counted after 2 days incubation at 37°C.

### Statistical Analysis

All statistical analyses were conducted using GraphPad Prism 7.0 (GraphPad Software, Inc). Comparisons between multiple groups were analyzed by one-way analysis of variance (ANOVA); survival curves were compared by log-rank (Mantel–Cox) test. A significant difference was defined as *p* < 0.05.

## Results

### Intranasal and Intrarectal Routes Need to Be Sequentially Combined for Optimal Induction of Rectum TRM

Conforming to the “common mucosal immune system” norm, several previous studies showed that intranasal administration of vaccine is able to elicit TRM in the vagina and intestine (25, 26). To test whether this is also the case for the induction of rectum TRM, we designed four different vaccination modalities with OVA as immunogen plus Cholera toxin (CT) as adjuvant being used in both prime and boost via either intranasal (IN) or intrarectal (IR) route and assessed their ability to induce rectal TRM in mice utilizing an adoptive transfer approach (Figure 1A). Specifically, we isolated CD45.1+ CD8+ T cells from OT-I mice, which carry a transgenic T cell receptor recognizing the OVA_257−264_ epitope and intravenously transferred them into wild-type CD45.2+ mice, which were subsequently subjected to immunization using one of the four regimens. Rectums and bronchoalveolar lavage fluids (BALs) were collected 4 weeks after the final boost and analyzed for CD45.1+CD8+ T cell response by flow cytometry. Compared to the IN + IR group, the frequency of these cells in rectum relative to total rectal CD8+ cells was dramatically reduced in the IN + IN group and to a slightly lesser extent in the IR + IR group while seeing an ~3-fold further increase in the IN + IR + IR group (Figures 1B,C). In contrast, except for the IR + IR group, all the groups showed robust elicitation of CD45.1+ CD8+ T cell subpopulation in the lung (Figures 1B,C). A similar trend was observed with respect to the absolute number of CD45.1+ OT-1 cells (Supplementary Figure 1). We also assessed the splenic OVA-specific T cell response by ELISPOT assay and found that such response was induced by all four treatments with IN + IR + IR and IN + IN groups showing a comparable level that was moderately higher than the other two groups (Supplementary Figure 2). Thus, the intranasal prime–intrarectal boost strategy was capable of effectively engaging both local and systemic T cell response.

We next assessed the expression of CD69 and CD103, two surface markers characteristic of tissue residences. The majority of CD45.1+ CD8+ T cells in the rectum in the IN + IN group did not express CD69 or CD103, which was in sharp contrast to the other three groups, in which CD69+ CD103+ cells emerged as the dominant subset with the rest of cells mostly belonging to the CD69+ CD103− subset (Figures 1D,E). We also conducted an immunofluorescence assay to analyze the localization of CD45.1+ CD8+ T cells in the samples of rectum mucosa from the IN + IR group. These cells were mainly detected in both epithelium and lamina propria, corroborating their TRM identity (Supplementary Figure 3). Thus, the effective induction of rectal TRM requires a combination of intranasal prime and intrarectal boost, surprisingly setting apart from the case of the induction of vaginal TRM, where either route alone appears to be somewhat effective.

Vaccination through intranasal route can elicit both systemic and mucosal immunity. Thus, we examined whether intramuscular route, which majorly provoke systemic immune response, could also be employed for priming in our strategy. As judged by the relative level of CD45.1 cells among the total CD8+ T cells after vaccination, intramuscular priming performed significantly better than intrarectal priming, albeit somewhat lower than intranasal priming (Figure 1F). This result reinforced the concept that coupling a systemic priming with an intrarectal pulling is the key to induce rectum TRM. Given the higher efficacy of intranasal priming vs. intramuscular priming, we adhered to intranasal priming for further exploration of our vaccination strategy.

### Intranasal Prime and Intrarectal Boost Strategy Raised Protective Rectal TRM in a Murine Model of Intrarectal LM-OVA Infection

**Figure 2:**
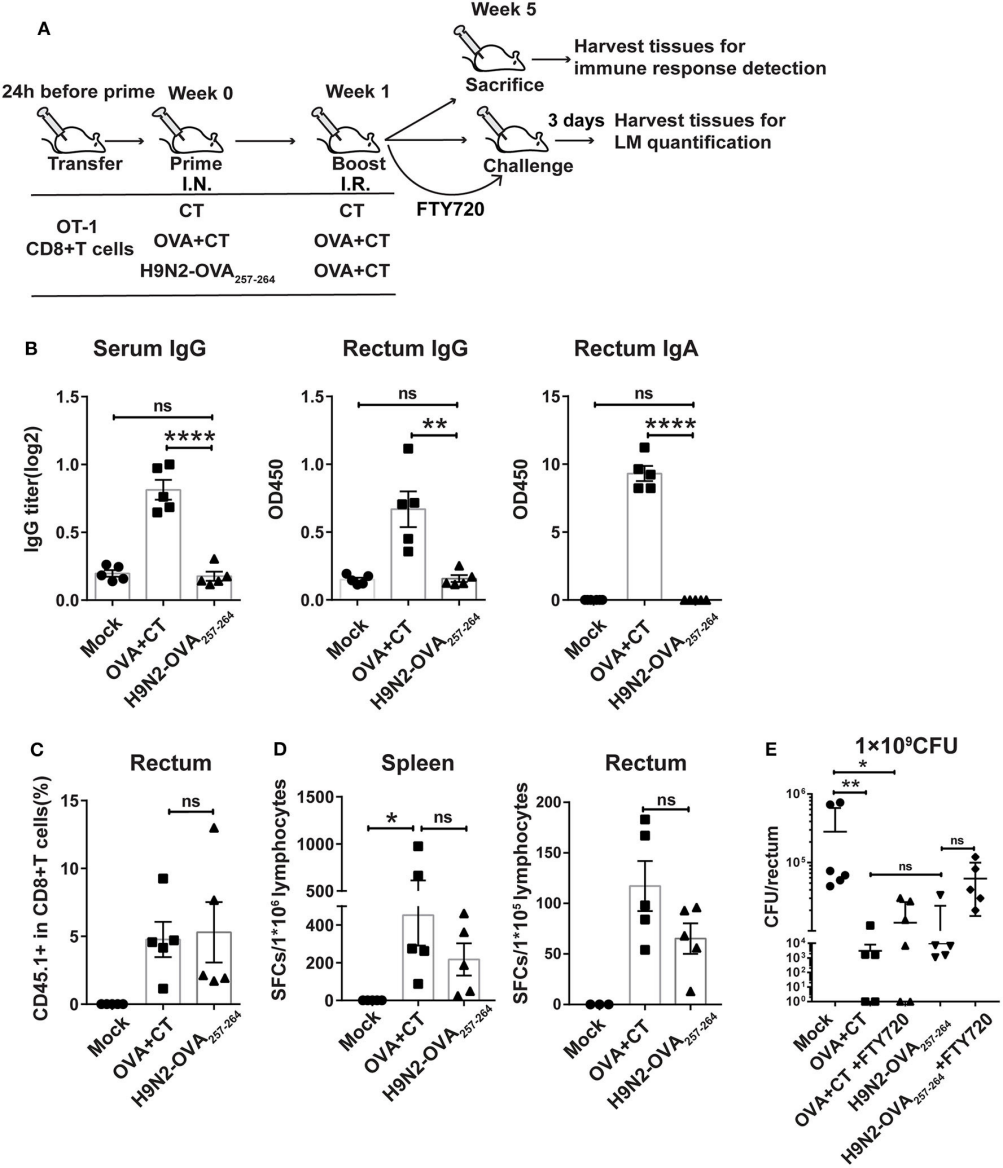
Protective effect mediated by intranasal prime and intrarectal boost strategy in a murine model of intrarectal recombinant *Listeria monocytogenes* expressing ovalbumin (LM-OVA) infection. **(A)** Experimental scheme. C57 BL/6 mice were intravenously transferred with OT-I cells (CD45.1+ CD8+) 1 day before intranasal priming with either a low-pathogenic H9N2 influenza virus expressing OVA_257−264_ epitope (H9N2-OVA_257−264_) or OVA protein plus CT as adjuvant (OVA + CT) and then boosted 7 days later with intrarectal application of OVA + CT. The mock group was primed and boosted with CT. At 4 weeks after the completion of immunization, the mice were either sacrificed for analyzing immune response or intrarectally challenged with 1 × 10^9^ CFU of LM-OVA. A subgroup of OVA + CT and H9N2-OVA_257−264_ vaccinated mice were daily treated with FTY720 intraperitoneally at a dose of 20 μg/mouse starting from 3 days before challenge and lasting throughout the course of LM-OVA challenge. All challenged mice were sacrificed 3 days after challenge for the detection of bacterial loads in the rectum. *n* = 5 for each experimental group. **(B)** OVA-specific antibody response in the serum and rectum homogenate assessed by ELISA. **(C)** Frequencies of CD45.1+ CD8+ T cells in total CD8+ T cells in the rectum analyzed by flow cytometry. **(D)** OVA-specific CD8+ T cell response in the spleen and rectum. Isolated lymphocytes were stimulated with OVA_257−264_ peptide, and then, the interferon-gamma (IFN-γ) secreting cells were quantified by ELISPOT assay. **(E)** Bacterial loads in the rectum. Bars represent mean ± SEM. *p < 0.05, **p < 0.01, ****p < 0.0001, ns: *p* > 0.05. Data are representative of three independent experiments.

To assess the protectiveness of rectum TRM elicited by the above intranasal prime–intrarectal boost vaccination strategy, we developed a murine model of LM-OVA (recombinant *L. monocytogenes* expressing ovalbumin) infection. This model was chosen because previous studies demonstrated that vaccination-mediated containment of LM-OVA is largely attributed to the action of CD8+ T cells (27, 28). Besides the IN + IR vaccination regimen as described above, we included a second regimen, which contains the same boost but the prime was changed to intranasal inoculation of a low-pathogenic H9N2 influenza virus (H9N2-OVA_257−264_) engineered to carry the OVA_257−264_ resides—the major histocompatibility complex (MHC) class I-restricted immunodominant epitope of OVA recognized by the adoptively transferred OT-I cells (Figure 2A). H9N2-OVA_257−264_ infection is thus supposed to only elicit CD8+ T cell response but not antibody response against OVA. Four weeks after the last immunization, mice were either sacrificed for tissue collection to assess immune response or subjected to rectal challenge with a non-lethal dose of LM-OVA. To differentiate the contribution of rectal TRM vs. systemic T cells to vaccine-induced protection, a subgroup of H9N2-OVA_257−264_-immunized mice were treated with FTY720, which blocks the egress of circulating memory via inhibition of S1P, starting the day before challenge until the animals were sacrificed for viral load determination (Figure 2A).

We first evaluated the OVA-specific antibody response in the serum and rectum lavage by ELISA. As expected, the OVA + CT group showed significantly higher OVA-specific antibody response in the two compartments than both H9N2-OVA_257−264_ group and mock group (Figure 2B). Next, we examined the presence of OT-I cells in the rectum by flow cytometry analysis. The induction of rectal CD45.1+ cells did not occur in the mock group but were readily detected in both vaccination groups, with their frequency relative to total rectal CD8+ cells being ~5% for both groups (Figure 2C). This was consistent with measurement by ELISPOT assay, showing comparable OVA-specific T cell responses between the two groups in both the spleen and rectum (Figure 2D). In the LM-OVA challenge study, we evaluated the vaccine effectiveness by measuring the rectal bacterial load 3 days after intrarectal LM-OVA challenge. The result showed ~a 20-fold reduction in the bacterial burden of the rectum in the two immunized groups compared to the mock group (Figure 2E). The similar protection afforded by the two immunization regimens juxtaposed with little induction of OVA-specific antibody response in the H9N2-OVA_257−264_ group led us to infer that vaccination-induced CD8+ T cells were primarily responsible for the conferred protection. This also validated the suitability of LM-OVA infection model for studying T-cell-based vaccines. Within the FTY720-treated subgroup of H9N2-OVA_257−264_ vaccinated animals, the rectal bacterial load was greater than the FTY720-untreated vaccination group but was still 4-fold lower than the mock control (Figure 2E). FTY720-treated, OVA + CT vaccinated animals also showed effective containment of LM-OVA, with a rectal bacterial load only slightly higher than that of the FTY720-untreated counterparts (Figure 2E). Thus, both systemic and rectum-lodged CD8+ T cell response appeared to mediate the IN + IR regimen-induced protection against intrarectal LM-OVA infection.

### Optimizing the Regimen Through an Exploration of Antigen Forms and Adjuvants

**Figure 3:**
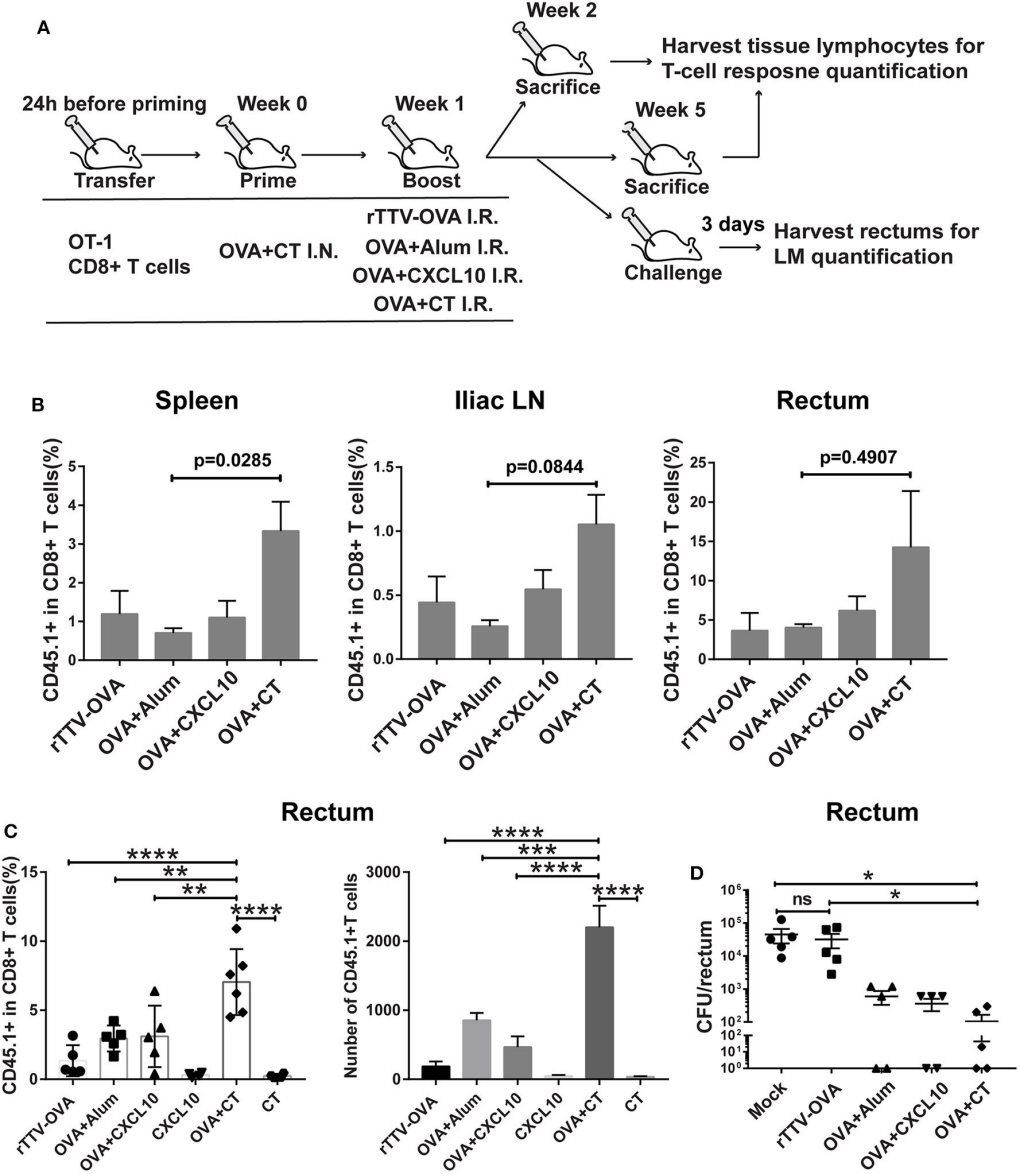
Optimizing intrarectal boost step through the exploration of antigen forms and adjuvants. **(A)** Experimental schedule and vaccination groups. rTTV-OVA represents recombinant Tiantan vaccinia viral vector expressing OVA, and the dose used was 2 × 10^6^ PFU. **(B)** Pooled data showing the frequencies of CD45.1+ CD8+ T cells as percentages of total CD8+ T cells in the spleen (left), iliac node (middle), and rectum (right) of vaccinated mice isolated at 1 week after the boost, *n* = 4 per group. **(C)** Relative abundance of rectal CD45.1+ CD8+ T cells measured by flow cytometry at 4 weeks after the boost, *n* = 4–6 per group. **(D)** The vaccinated mice were intrarectally infected with recombinant *Listeria monocytogenes* expressing ovalbumin (LM-OVA) at 4 weeks after the boost, and rectums were isolated 3 days postinfection for assessment of bacterial loads, *n* = 5 per group. Bars showed the mean ± SEM; *p < 0.05, **p < 0.01, ****p < 0.001, ****p < 0.0001, ns: *p* > 0.05. Data are representative of three independent experiments.

We then attempted to further improve our immunization strategy by trying different antigen forms and adjuvants for the intrarectal boost step in the context of the same intranasal prime. The vaccination groups and the experimental schedule are shown in Figure 3A. Our interest in rTTV-OVA stemmed from our previous success in using rTTV-based vaccines to raise both mucosal and systemic immune responses (29). Alum has been widely used in clinical vaccines with documented safety (30). The tryout of chemokine (C-X-C motif) ligand 10 (CXCL10) was based on a review of the literature demonstrating that topical application of chemokine is an effective approach to recruit (pull) activated CD8+ cells from circulation to the targeted peripheral tissue, e.g., vagina and skin (20, 31).

Our temporal analyses of vaccine-raised, antigen-specific CD8+ T cell response consistently revealed a profound impact of intrarectal boost formulation. At 1 week postboost, when the effector phase is proceeding, the OVA + CT boost raised higher levels of CD45.1+ cells in the spleen, iliac lymph node, and rectum than the other three boosts (Figure 3B). The superiority of OVA + CT boost was also displayed in the formation of rectal TRM, which was detected at 4 weeks after the boost. Among the four boost modalities, rTTV-OVA is the least effective in inducing rectal TRM, with CD45.1+ cells accounting for only 2% of total CD8+ T cells. The OVA + alum and OVA + CXCL10 groups showed similarly better induction of rectal TRM compared to the rTTV-OVA group, although still ~2-fold lower than the OVA + CT group (*p* = 0.0091, *p* = 0.0099) (Figure 3C). We also analyzed the absolute number of rectal CD45.1+ CD8+ T cells in the four groups and found it to be consistent with the frequency data (Figure 3C). As control, individual administration of CT and CXCL10 alone failed to induce rectum TRM, excluding the presence of antigen-independent mechanism (Figure 3C). The superior capability of the OVA + CT boost to augment both systemic and rectum CD8+ T cells is consistent with the notion that CD8+ T cells entering secondary lymph nodes can leave for either periphery site of infection or systemic circulation after activation by antigen-loaded APCs.

We further determined whether antigen/adjuvant selection in the boost step can affect *in vivo* protective efficacy. The protection of vaccinated mice against non-lethal rectal LM-OVA challenge, as assessed by a reduced viral load of the rectum, showed a positive correlation with the rectal TRM level, with the OVA + CT boost leading to the strongest protection (Figure 3D)_._ Collectively, these investigations supported, among all the rectum boost formulations tested, that protein–CT combination performs best for our prime–boost strategy.

### The Intranasal Prime–Intrarectal Boost Strategy Elicits Rectum TRM From the Endogenous T Cell Repertoire to Afford Protection Against Rectal LM Infection

**Figure 4:**
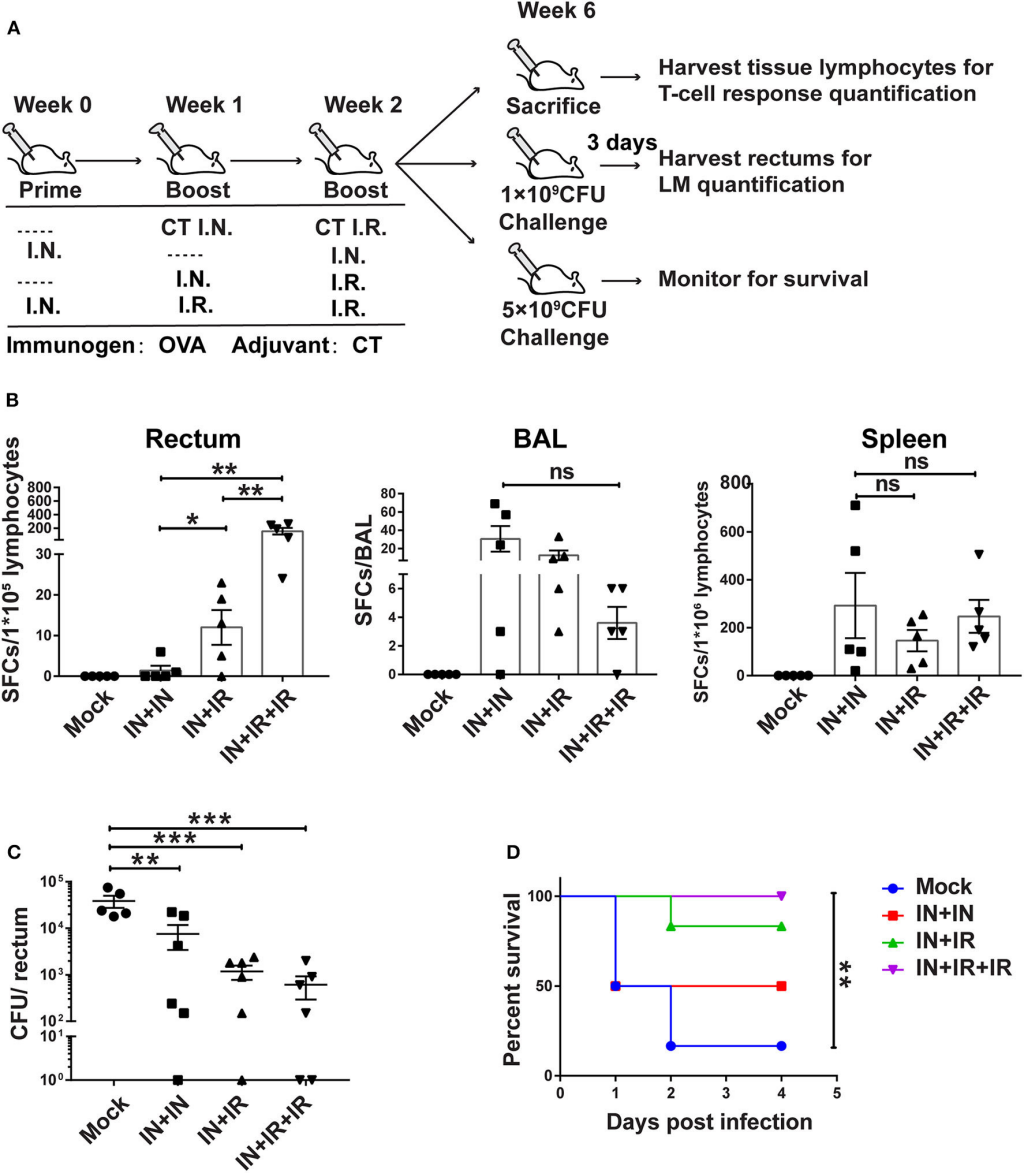
The intranasal prime–intrarectal pull strategy was effective in generating rectal TRM cells from endogenous T cell repertoire and protecting against rectal challenge in wild-type mice. **(A)** Schematic representation of experimental protocol and immunization groups. Mice were immunized with ovalbumin + cholera toxin (OVA + CT) in three groups: IN + IN, IN + IR, IN + IR + IR; mock group used only CT for both intranasal prime and intrarectal boost. The immunized mice were subjected to rectal infection with LM-OVA at 4 weeks after immunization or sacrificed 1 day earlier to collect tissue for assessment of cellular response. **(B)** Tissue-localized OVA_257−264_-specific, interferon-gamma (IFN-γ)-secreting T cell response after vaccination. Lymphocytes were isolated from the rectum, BAL, and spleen of vaccinated animals, and their response to stimulation with OVA_257−264_ was measured by IFN-γ-ELISPOT assay. **(C,D)** Vaccine-mediated protection against rectal LM-OVA challenge. The vaccinated mice were intrarectally infected either with non-lethal dose (1 × 10^9^ CFU) of LM-OVA followed by the determination of the bacteria load in the rectum at 3 days after challenge **(C)** or with a lethal dose (5 × 10^9^CFU) of LM-OVA to measure the survival **(D)**, *n* = 5 for **(B)**, *n* = 6–7 for **(C,D)**. Bars are the mean ± SEM. *p < 0.05, **p < 0.01, ***p < 0.001, ns: *p* > 0.05. Data are representative of three independent experiments.

Our adoptive transfer mouse model demonstrated that our novel intranasal prime–intrarectal boost strategy was capable of eliciting immunogen-specific rectal TRM. We next implemented this strategy in a natural context, i.e., in the absence of adoptive transfer. We immunized mice with OVA + CT following four different schemes (Figure 4A). Each experimental group was split into one non-challenged group and two challenged subgroups. For non-challenged subgroups, mice were sacrificed 4 weeks after the last boost to isolate the rectum, BAL, and spleen for analysis of OVA-specific T cell response. Consistent with the data mentioned above from the adoptive transfer model, the OVA-specific T cell response in the rectum, expressed as the frequencies of OVA_257−264_-specific IFN-γ spot forming cells (SFCs) measured by ELISPOT, was significantly higher in the IN + IR group than in the IN + IN group (*p* = 0.0476). The addition of the second IR boost resulted in an ~10-fold further increase in the number of SFCs (*p* = 0.0019) (Figure 4B, left panel). In sharp contrast, all three immunization groups showed significant induction of OVA_257−264_-specific T cells in BAL and spleen compartments as compared to the mock group (Figure 4B, middle and right panel). Although not statistically significant, it is notable that the magnitude of OVA_257−264_-specific T cell response in BAL displayed an inverse correlation with that in the rectum.

For the two challenged subgroups, mice were intrarectally infected with non-lethal dose or lethal dose LM-OVA 4 weeks after immunization. The outcomes of infection were subsequently evaluated by measurements of rectum bacteria load and survival, respectively. The evaluation revealed the importance of intrarectal boost. When facing a non-lethal LM-OVA challenge, the IN + IR group contained the bacteria significantly more effectively than the IN + IN group, and the addition of second intrarectal boost further improve such containment (mock vs. IN + IN: *p* = 0.0036; mock vs. IN + IR: *p* = 0.0006; mock vs. IN + IR + IR: *p* = 0.0005) (Figure 4C). The same trend was observed in the case of lethal LM-OVA challenge: 4 days after the challenge, the IN + IR + IR group all survived, and the IN + IR group showed a survival rate of 80%, which was higher than the 50% survival observed with the IN + IN group. In contrast, the survival rate of the mock group was significantly lower (10%) (Figure 4D). The positive correlation between the induction level of rectal TRM and the protection efficacy reinforced our view that establishing a potent rectal TRM response is central to our strategy to protect from intrarectal infection.

### Circulating Recently Activated T Cells Serve as the Primary Contributor to Rectum TRM Formation

**Figure 5:**
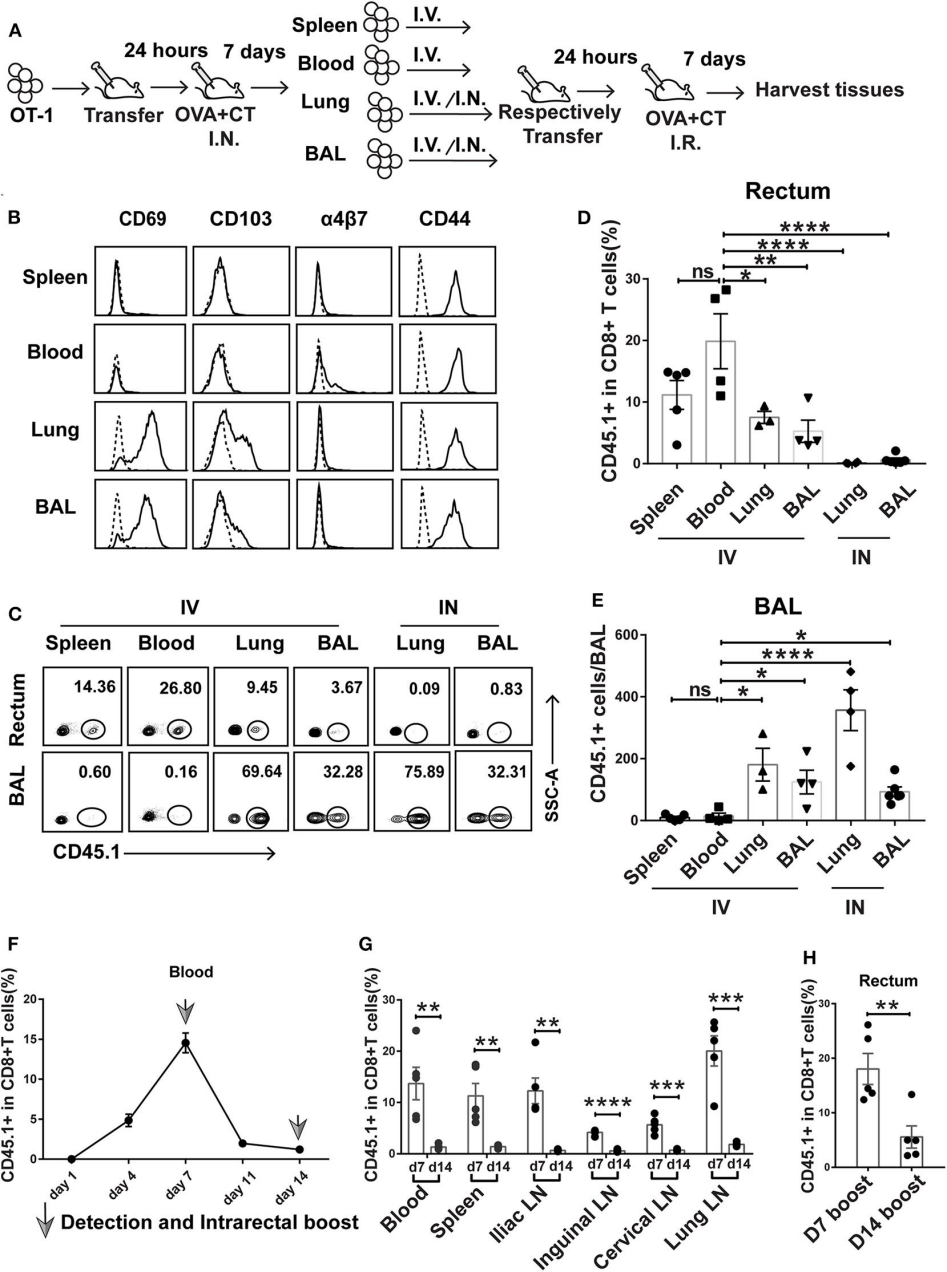
Prime-activated circulating CD8+ T cells served as the primary contributor to rectum-resident memory T cell (TRM) formation. **(A)** Schematic representation of a successive adoptive transfer approach to tracking the origin of rectum TRM. Native C57 BL/6 mice were intravenously transferred with OT-I (CD45.1+ CD8+) T cells 1 day before intranasal vaccination with OVA + CT. After 1 week, the CD45.1+CD8+ T cells were isolated from the spleen, blood, lung, and BAL of the primary recipients and then individually transferred into naive C57 BL/6 mice either intravenously (IV) or intranasally (IN) (1 × 10^5^ cells per mouse). At 24 h posttransfer, the secondary recipient mice were intrarectally inoculated with OVA + CT and sacrificed 1 week later to analyze the tissue distribution of CD45.1+ CD8+ T cells. **(B)** Representative flow cytometry profiling of surface marker expression on the second generation of CD45.1+ CD8+ T cells isolated from the primary recipient. **(C–E)** Determination of tissue distribution of CD45.1+ CD8+ T cells in secondary recipient mice by flow cytometry. Representative flow cytometry plot was shown in **(C)**, and the cumulative results depicting the frequency of CD45.1+ CD8+ T cells among total CD8+ cells in the rectum and BAL were displayed in **(D)** and **(E)**, respectively. **(F,G)** Dynamics of circulating T cell response after intranasal priming with OVA + CT. The CD45.1+ CD8+ OT-I cells were transferred to naive C57BL/6 mice. After 24 h, the recipient mice were intranasally inoculated with OVA + CT, and the blood was subsequently isolated at the indicated time for the determination of the frequency of OT-I cells (expressed as percentage of the total CD8+ T cells) by flow cytometry, shown in **(F)**. The distribution of OT-I cells in secondary lymph tissues was also determined on day 7 (d7) and day 14 (d14), shown in **(G)**. **(H)** The effect of extending the prime–boost interval from 7 to 14 days on the induction of rectal TRM. The vaccination regimen used for the D7 and D14 groups were the same as that for the IN + IR group described in Figure 1 except that, for D14 group, the interval between IN prime and IR boost was extended to 14 days. The tissues were collected 1 week after the boost. Shown are cumulative results comparing the frequencies of CD45.1+ cells, expressed as percentage of the total CD8+ T cells, in the rectum between the two groups. *n* = 3–5 per group. Bars represent the mean ± SEM. *p < 0.05, **p < 0.01, ***p < 0.001, ****p < 0.0001, ns: *p* > 0.05. Data are representative of three independent experiments.

To gain insight into the ontogeny of rectum TRM, we designed a successive adoptive transfer experiment (Figure 5A). We first adoptively transferred OT-I cells into naive wild-type mice, which were subsequently intranasally immunized with OVA + CT. At day 7 postimmunization, the CD8+ cells derived from transferred OT-I cells were separately isolated from the spleen, blood, lung, and BAL of the recipient mice by flow cytometry and retransferred intravenously or intranasally into naive wild-type mice. The secondary recipient mice were then intrarectally immunized with OVA + CT 1 day later, and after 7 days, they were sacrificed to collect rectum tissue for detection of the second generation of transferred OT-I cells.

Flow cytometry analysis of the first generation of OT-I donor cells from the four compartments reveals shared and distinct phenotypes. All the cells express CD44 irrespective of origin, confirming their activation status. In contrast, only those from the lung and BAL express CD69 and CD103, indicative of a tissue-residency phenotype, whereas the expression of α4β7 integrin, a homing receptor selectively expressed on gastrointestinal homing CD8+ cells, is restricted to blood-derived donor cells (Figure 5B). The first generation of OT-I donor cells, when retransferred to naive mice, responded differently to rectal OVA + CT immunization, depending on their history of tissue residency. In the case of retransferring via the intravenous route, the blood-derived donor cells were the readiest for rectal recruitment, followed by spleen-derived cells, with the average percentage of CD45.1+ cells in the total rectal CD8+ cells being about 20 and 12%, respectively. Lung- and BAL-derived donor cells showed significantly less rectum lodgment, with the average of such relative frequency of CD45.1+ cells decreased to 5–8% (Figures 5C,D). Contrastingly, these donor cells but not cells of blood or spleen origin showed significant lung distribution (Figures 5C,E). This default recruitment without local antigen presentation was more clearly displayed when the lung- and BAL-derived donor cells were intranasally instilled, resulting in effective retention in the lung and essentially no detectable migration to the rectum (Figures 5C–E). Thus, we conclude that among the activated CD8+ T cells induced by intranasal priming, those remaining in the circulation are most likely primary target of subsequent intrarectal boost for recruitment to the rectum, whereas those already partitioned into the lung tissues, despite maintaining at least a partial rectum-relocalization capacity, are more likely to stay in their lung niches.

Circulated prime-activated T cells are not a stagnant population. Instead, they undergo continuous changes over the course from the effector phase to the memory phase. Previous studies found that newly activated T cells have an intrinsic capability to migrate to gastrointestinal compartments, but such capability progressively diminishes over time (32). Thus, we speculate that changing the interval between intranasal priming and intrarectal boosting might influence the outcome of vaccination. To explore this speculation, we adoptively transferred OT-I cells to naive mice and monitored the temporal changes of the magnitude of circulating activated T cells after intranasal administration of OVA + CT. A biphasic curve was observed with a peak at day 7, which is in line with the activated T cell experiencing from clonal expansion to massive contraction toward memory development (Figure 5F). In connection, extending prime–boost interval from day 7 to 14 was accompanied by a sharp decrease in the frequency of CD45.1+ cells relative to total CD8+ T cells in the secondary lymphoid tissues (Figure 5G). Such extension also significantly attenuated the generation of rectal TRM (Figure 5H). Thus, it appeared that the 7-day prime–boost interval, which sets the primed T cells at the effector phase, is a critical parameter of our vaccination strategy.

## Discussion

TRMs reside in various tissues and serve as sentinel leading the first-line defense against periphery invasion of pathogens. In this study, we focused on exploring the immunization means to induce a protective rectum TRM response, which might hold the key to a successful HIV vaccine. Based on a murine model of LM-OVA infection in combination with adoptive T cell transfer approach, we identified a systemic prime–intrarectal pull immunization strategy as the optimal strategy to induce protective rectal TRMs. We provided evidence that this strategy engaged both systemic and rectum-resident T cell response to effectively protect vaccinated mice from LM infection. The subsequent studies demonstrated the importance of prime–pull interval in the effective induction of rectal TRM, revealing the circulating recently prime-activated effector T cells as the primary pullable precursors for rectal TRM formation.

The initial rationale underlying our exploration of intranasal route for the generation of rectum TRMs was based on the concept of “common immune compartment,” which posits a likeness between immunity raised in different mucosal site, thus implicating that the induction of immune response in a given mucosal site can be achieved by vaccination at remote mucosal site(s) (33, 34). For instance, it was shown previously that vaginal TRMs could be significantly elicited by intranasal immunization alone (19, 35). The failure of intranasal immunization alone in raising rectal TRMs indicates strongly that, from a TRM perspective, the vagina is likely to be closer to the lung than the rectum. Given the tissue residence feature of TRMs, it stands to reason that a local delivery may perform better than a systemic delivery for their induction. Several previous studies indeed showed that topical administration of vaccine was able to elicit TRMs in various tissues, including the lung (36), skin (37)„ vagina (25, 26), and rectum (16, 17). In contrast, we demonstrated here that intrarectal vaccination only works when coupling to preceding intranasal vaccination to generate rectal TRM. It should be noted that previous studies on the intrarectal route mainly made a comparison to parental route but not to a combined regimen as shown in this study (17). The inefficacy of intrarectal antigen application in eliciting *in situ* immune response is consistent with the tolerogenic nature of gastrointestinal microenvironment and the scarcity of circulating antigen-recognizing naive T cells. The fact that a prime through intramuscular route could also work in our sequential strategy (Figure 1F) highlighted that the establishment of a systemic T cell response was the key for intranasal prime to facilitate the intrarectal pull in establishing rectal TRM. By enriching the antigen-specific T cells ready for the recruitment, a prior systemic immunization allows the intrarectal immunization to be more fruitful for the induction of rectum TRM. The same paradigm has been revealed in the studies of vaccination strategy to establish long-standing lung immunity, where the potentially most effective way to induce lung TRM is also found to be a sequential combination of systemic priming and local pulling (38–40). Whether such resemblance reflects the intrinsic similarity between rectal and lung TRM needs further investigation.

The measure by which activated T cells are recruited from the systemic circulation to target tissues is a major determinant of success in prime–pull immunization strategy. One measure previously identified is topical chemokine application, which showed success in promoting the trafficking of activated CD8+ T cells to the genital tract (20). In contrast, we showed that CXCL10 was less effective than CT as an adjuvant in the intrarectal pull step. A major mechanism behind CT's adjuvant activity is stimulating antigen-presenting cells for enhanced antigen presentation (24, 41, 42). This property of CT is consistent with our notion that the rectum homing of primed CD8+ T cells is mainly licensed by ligation to locally presented cognate antigens. The importance of the antigen-dependent mechanism is also recognized by the two most recent reports that investigated prime–pull strategy to induce lung (43) and vaginal (44) TRMs, respectively. We propose that although TRM recruitment can be attained by both antigen-dependent and antigen-independent mechanisms, the former is generally more potent and thus more suitable for the induction of TRMs, particularly in those tissues where the microenvironment is less permissive to this induction, the rectum as an instance. Accordingly, a powerful local antigen pull might be able to change the prime-imprinted tissue destination of CD8+ T cells, as seen in the new IN prime–IR boost strategy. The poor performance of the TTV-based pull approach in our investigation may be caused by inefficiency in engaging either or both recruitment mechanisms.

The potential analyses revealed that circulating activated CD8+ T cells are most amenable to antigen-dependent rectum recruitment. Interestingly, primed CD8+ T cells derived from lung or lung airway (BAL) also showed potential for rectal recruitment, albeit less effective than systemic CD8+ T cells (Figure 5D). However, the latter cells also show voluntary retention to the lung when intranasally transferred. Thus, it remains to be determined whether these cells take a one-way trip to the lung or they can re-enter into the circulation to participate in immune response in other tissues. It is noteworthy that a critical aspect of the potential assay is to ensure the purity of the isolated tissue-residing CD8+ T cells without contamination of circulating CD8+ T cells, which is of particular concern for lung TRM. We had addressed this concern by intravenous staining, in which an anti-CD8 labeled antibody was intravenously injected prior to lung TRM isolation. The analyses showed that ~35% of the isolated CD8+ T cells were positive for intravenous antibody (unpublished data). However, these positively stained cells potentially represent residing CD8+ T cells associated with lung capillary network, according to a previous report from Masopust's group (45). This possibility was corroborated by our finding that the isolated lung CD8+ cells were distinct from blood T cells in terms of a vast majority being CD69 positive (Figure 5B). Thus, we were convinced that CD8+ T cells isolated from the lung by our method indeed were primarily lung-residing T cells with a subpopulation probably present within the capillary.

During the optimization of our prime–pull strategy, we found that the prime–pull interval significantly influences the efficacy of TRM generation. Increasing the interval from 7 to 14 days remarkably reduced rectal TRM induction. Although we cannot exclude the possibility that the level of circulating antigen-specific T cells contributes to such interval effect, this observation is in agreement with previous finding that the trafficking capacity of activated circulating T cells positively correlates with their freshness, probably due to the transient upregulation of α4β7, the homing receptor for MAdCAM-1 expressed on the endothelial cell surface (32). Interestingly, several most recent studies applying prime-and-pull strategy to induce vaginal TRM used a prime–pull interval up to 3 or 4 weeks (44, 46), indicating the generation of TRM from prime-established memory pools. Although newly activated effector T cells have a clear advantage over memory T cells in terms of size of the population, the effect of length of the interval between prime and pull on TRM generation is worth future investigations.

We demonstrated in two experimental settings, i.e., mice receiving adoptively transferred OT-I cells and wild-type mice, in which the IN prime–IR pull strategy afforded significant protection against the intrarectal LM-OVA challenge. Our use of the H9N2-OVA_257−264_, which carries a single OVA-specific T cell epitope, validated LM-OVA as a model pathogen to study antigen-specific T cell response. Consistent with its nature in terms of the encoded OVA epitope, priming with H9N2-OVA_257−264_ led to a profoundly reduced OVA-specific antibody response when compared to the priming with OVA + CT (Figure 2B). In contrast, the two different primings resulted in comparable levels of OVA-specific CD8 T cell response in both the spleen and rectum. In connection, they achieved similar control of LM-OVA. Thus, despite the lack of a final proof with a CD8+ T cell depletion experiment, our results were in line with the pivotal role of CD8+ T cell response in the anti-LM-OVA immunity. On the other hand, the superiority of IN + IR regimen over the IN + IN regimen, which failed to induce rectal TRM despite even higher systemic CD8+ T cell response, highlights the importance of rectal TRM in protection. That the protective efficacy of IN + IR regimen was compromised but not abolished by FTY720 treatment further indicated that rectal TRM is essential, but not sufficient, for a full protection. It should be noted that, although our FTY720 treatment very effectively (~98%) inhibited the exit of CD8+ T cells from secondary lymphoid tissues, the small percentage of escaped, circulation-entering CD8+ T cell might participate in the protection against LM-OVA. However, taking into account the level of protection conveyed by the IN + IN regimen (solely from systemic T cell response) vs. the IN + IR regimen, we have reason to think that, even if such participation did exist, the rectal TRM remains to be the main force underlying the anti-LM-OVA immunity observed upon FTY720 treatment. Our result is consistent with the notion that reactivation of TRM cells may help recruit recirculating memory T cells to the port of pathogen entry, together mounting a protective response (47). Thus, IN + IR regimen affords a crucial advantage over IN + IN and IR + IR regimens by engaging both systemic and rectum-resident memory.

In sum, we presented in this study a novel systemic prime–rectal pull vaccination strategy centered on the establishment of rectum TRM cells and demonstrated that its administration protected mice from rectal LM-OVA infection through the combined action of circulating and rectum-resident memory T cells. As this strategy is explored aiming to tackle HIV virus, it will be exciting to test and optimize this strategy in the setting of NHP SIV/SHIV infection model, which will help answer the ultimate question of whether embracing both TRM and systemic arms of human CD8+ T cell immunity is sufficient for protection from HIV.

## Data Availability Statement

The raw data supporting the conclusions of this article will be made available by the authors, without undue reservation.

## Ethics Statement

The animal study was reviewed and approved by the Institutional Animal Care and Use Committee of Shanghai Public Health Clinical Center.

## Author Contributions

JX and XZ conceived, designed, and supervised the study. QH helped design the study, performed the majority of the experiments, analyzed the data, and wrote the original draft. CZ helped design the study. LJ and KC helped conduct some experiments. XX, LZ, SZ, XD, YH, TQ, XJ, and CZ contributed to data analysis. MZ contributed experimental materials. CZ, JX, and XZ edited the manuscript. All authors reviewed and approved the manuscript.

## Conflict of Interest

Shanghai Public Health Clinical Center has filed a Chinese patent application (No. 201910874907.3), with JX, XZ, and QH being listed as inventors. The remaining authors declare that the research was conducted in the absence of any commercial or financial relationships that could be construed as a potential conflict of interest.
