# Supplementary material for: A Systemic Prime–Intrarectal Pull Strategy Raises Rectum-Resident CD8+ T Cells for Effective Protection in a Murine Model of LM-OVA Infection
Source: Front Immunol. 2020 Sep 24;11:571248. doi: 10.3389/fimmu.2020.571248 (PMC7541937; doi:10.3389/fimmu.2020.571248)
Supplement: Supplementary file 1 [file Data_Sheet_1.PDF]

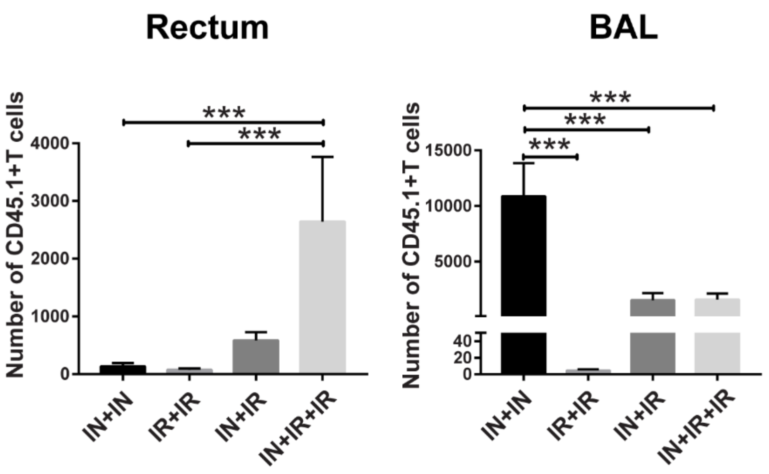

Supplementary Figure 1. Comparison between different immunization regimens in the absolute number of CD45.1+ CD8+ T cells induced in the rectum (left panel) and in the BAL (right panel). The pooled frequency data were shown in Figure 1. Bars represent the mean ± SEM, ***p<0.001.

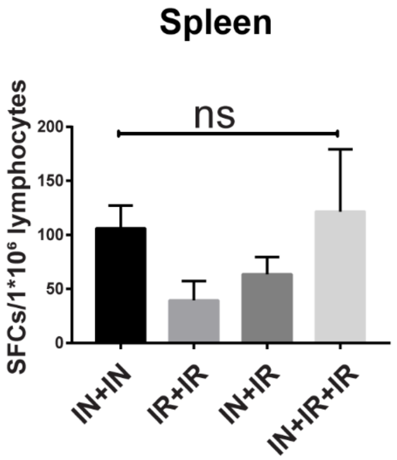

Supplementary Figure 2. Impact of administration route of priming and boost on systemic antigen-specific T cell response. The four immunization groups were the same as described in Figure 1. The isolated lymphocytes were stimulated with $OVA_{257\text{-}264}$ peptide, and then the IFN-γ secreting cells were quantified by ELISPOT assay. Bars represent the mean ± SEM, ns: p > 0.05.

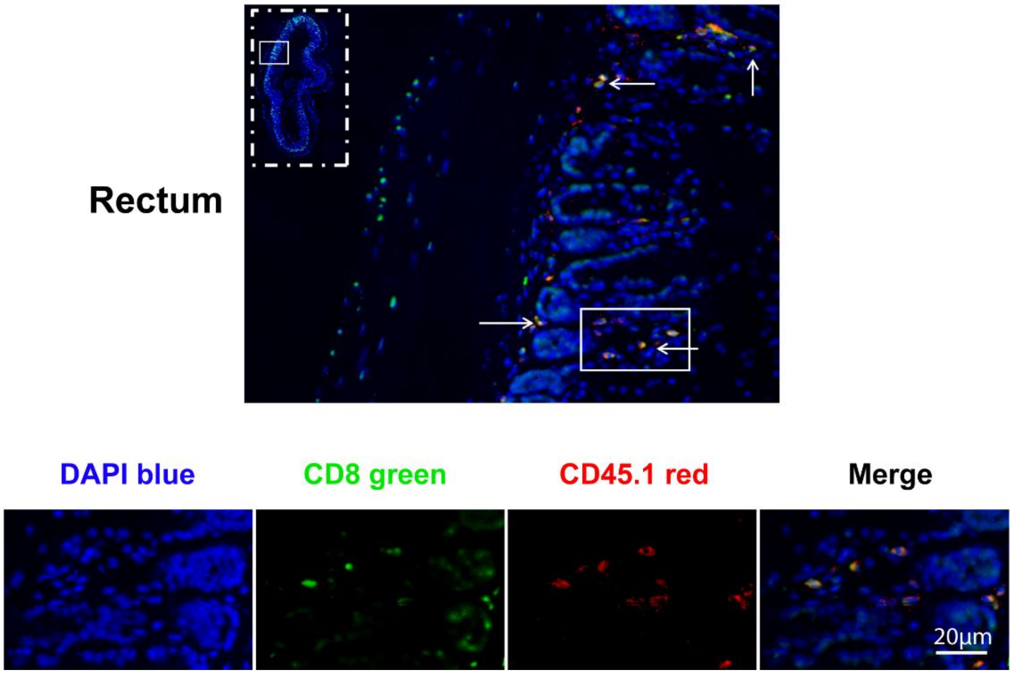

Supplementary Figure 3. Localization of TRM raised by IN+IR regimen in the rectal mucosa. Rectum tissue sections were prepared from the same IN+IR group shown in Figure 1 at 4 weeks post vaccination, and co-stained for DAPI (blue), CD8 Ab(green), and CD45.1(red). Frames mark the areas that were explored for magnified images, and arrows indicate the detected individual CD8/CD45.1 double positive cells. Images are representative of 3 animals.
